# Transthoracic 3D echocardiographic left heart chamber quantification in patients with bicuspid aortic valve disease

**DOI:** 10.1007/s10554-017-1192-1

**Published:** 2017-06-19

**Authors:** Allard T. van den Hoven, Jackie S. Mc-Ghie, Raluca G. Chelu, Anthonie L. Duijnhouwer, Vivan J. M. Baggen, Adriaan Coenen, Wim B. Vletter, Marcel L. Dijkshoorn, Annemien E. van den Bosch, Jolien W. Roos-Hesselink

**Affiliations:** 1 Department of Congenital Cardiology, Erasmus MC, Room Ba-583a, P.O. Box 2040, 3000 CA Rotterdam, The Netherlands; 2 Department of Radiology, Erasmus MC, Rotterdam, The Netherlands; 3 Department of Cardiology, Erasmus MC, Rotterdam, The Netherlands; 4 Department of Cardiology, UMC Radboud University Medical Center, Nijmegen, The Netherlands

**Keywords:** 3D echocardiography, Fully automated volumetric chamber quantification, Computed tomography, Left heart model, Bicuspid aortic valve

## Abstract

Integration of volumetric heart chamber quantification by 3D echocardiography into clinical practice has been hampered by several factors which a new fully automated algorithm (Left Heart Model, (LHM)) may help overcome. This study therefore aims to evaluate the feasibility and accuracy of the LHM software in quantifying left atrial and left ventricular volumes and left ventricular ejection fraction in a cohort of patients with a bicuspid aortic valve. Patients with a bicuspid aortic valve were prospectively included. All patients underwent 2D and 3D transthoracic echocardiography and computed tomography. Left atrial and ventricular volumes were obtained using the automated program, which did not require manual contour detection. For comparison manual and semi-automated measurements were performed using conventional 2D and 3D datasets. 53 patients were included, in four of those patients no 3D dataset could be acquired. Additionally, 12 patients were excluded based on poor imaging quality. Left ventricular end-diastolic and end-systolic volumes and ejection fraction calculated by the LHM correlated well with manual 2D and 3D measurements (Pearson’s r between 0.43 and 0.97, p < 0.05). Left atrial volume (LAV) also correlated significantly although LHM did estimate larger LAV compared to both 2DE and 3DE (Pearson’s r between 0.61 and 0.81, p < 0.01). The fully automated software works well in a real-world setting and helps to overcome some of the major hurdles in integrating 3D analysis into daily practice, as it is user-independent and highly reproducible in a group of patients with a clearly defined and well-studied valvular abnormality.

## Introduction

Left atrial and ventricular volumes and ejection fraction are important diagnostic and prognostic parameters, widely used in daily practice. Indication for cardiac surgery in patients with valvular abnormalities such as bicuspid aortic valve disease, relies heavily on accurate left ventricular (LV) volume and function assessment. For many years, two-dimensional echocardiography (2DE) has been the most widely used modality for LV volumetric assessments; however, it is based on geometric assumptions which cause inaccuracy and the reproducibility remains suboptimal. Three-dimensional echocardiography (3DE) has largely overcome these drawbacks as it has the ability to visualize cardiac structures from any perspective, entailing an accurate quantitative and more reproducible evaluation of cardiac chambers. However, the use of 3DE in daily clinical practice has been hampered, because there is a learning curve for data acquisition, and 3D data analysis can be a time-consuming process, moreover there is need for experienced observers.

A fully automated and user-interference free algorithm may improve the feasibility of 3DE in daily clinical practice. Such a method has now been proposed in the new ‘Heart Model’ Software (LHM), which promises a rapid and accurate automated quantification of left atrial (LAV) and LV volumes and ejection fraction (LVEF). The feasibility and accuracy of the new “Heart Model” was recently compared to cardiac magnetic resonance imaging by Tsang et al. who concluded that this technique is strongly correlated with CMR, with a high reproducibility and short analysis time [1, 2]. However, it has not been reported whether this also applies to patients with valvular heart disease where high reproducibility and feasibility is very important.

Therefore, this study aims to assess feasibility and reproducibility of the ‘Heart model’ software in a prospective cohort study by comparing the results between echo and CT using conventional 2D, 2D-xPlane, 3D transthoracic echocardiography TTE (3DE) techniques in patients with a bicuspid aortic valve (BAV) with moderate to severe aortic valve stenosis or regurgitation.

## Methods

### Patients selection

Patients with a BAV who visited the outpatient clinic for regular follow-up between October 2014 and March 2016 were consecutively included into this prospective study. All patients underwent the full study protocol on the same day. The study protocol consisted of physical examination, electrocardiography (ECG), 2D and 3D echocardiography and a cardiac CT scan. The study was approved by the medical ethical committee of the Erasmus medical center and informed consent was given by all patients who participated in the study. Two protocols were used reference methods were used to compare the LFM data: in the first protocol 3DE was used as a reference standard, in the second protocol the LHM was compared to CT.

### Echocardiography

#### Image acquisition

Two experienced sonographers (J.S.M., W.B.V.) performed a standard two-dimensional transthoracic echocardiogram (2DE). All studies were acquired in the left lateral decubitus position, in harmonic imaging using an EPIQ7 ultrasound system (Philips Medical Systems, Best, the Netherlands) equipped with a x5–1 matrix-array transducer (composed of 3040 elements with 1–5 MHz) A non-foreshortened apical four-chamber view (A4C) and two-chamber view (A2C) were recorded with manual or electronic rotation (iRotate) followed by a focused LV, A4C and A2C image. From the focused LV-A4C a true perpendicular image view (A2C) was acquired with xPlane mode, in order to retrieve both views from the same heart-beat. This was repeated with the focus on the true long axis of the LA [3]. Real-time 3D-TTE was performed immediately after the 2D-TTE with the same ultrasound unit and transducer. A four-or six-beat full volume dataset of the LV and LA was acquired from the apical window during a single breath hold. Two extra datasets were acquired from the A4C view in the dedicated ‘Heart Model’ acquisition mode.

#### Image analysis

Analysis was performed by A.T.H. and J.S.M. All measurements were blinded to patient specific information. Before measurements were performed, the quality of each dataset was evaluated by both observers. Patients were excluded from further analysis in cases of poor image quality (e.g. poor endocardial visualization).

##### 2D echocardiography

LV end diastolic volume (LVEDV), LV end systolic volume (LVESV) and LVEF were calculate using the Simpsons bi-plane method of disk summation, as stated in the guidelines, from the standard A4C and A2C and apical xPlane images [4]. LAV was calculated using the area length method.

##### 3D echocardiography

Manual: LV volumes and LVEF were measured using commercially available software (QLab-3DQ, Philips medical systems). The user aligned the multiplanar view to obtain the true long axis of the LV in the A4C and A2C view. Landmarks were placed on the mitral annulus and apex. The endocardial border was traced automatically and adjusted manually where needed. For the LA volume, the true long-axis was aligned using the multiplanar mode in the end-systolic frame and the contour traced as mentioned above. The 3D dataset was scored feasible when the entire cardiac contour could be traced.

**Fig. 1:**
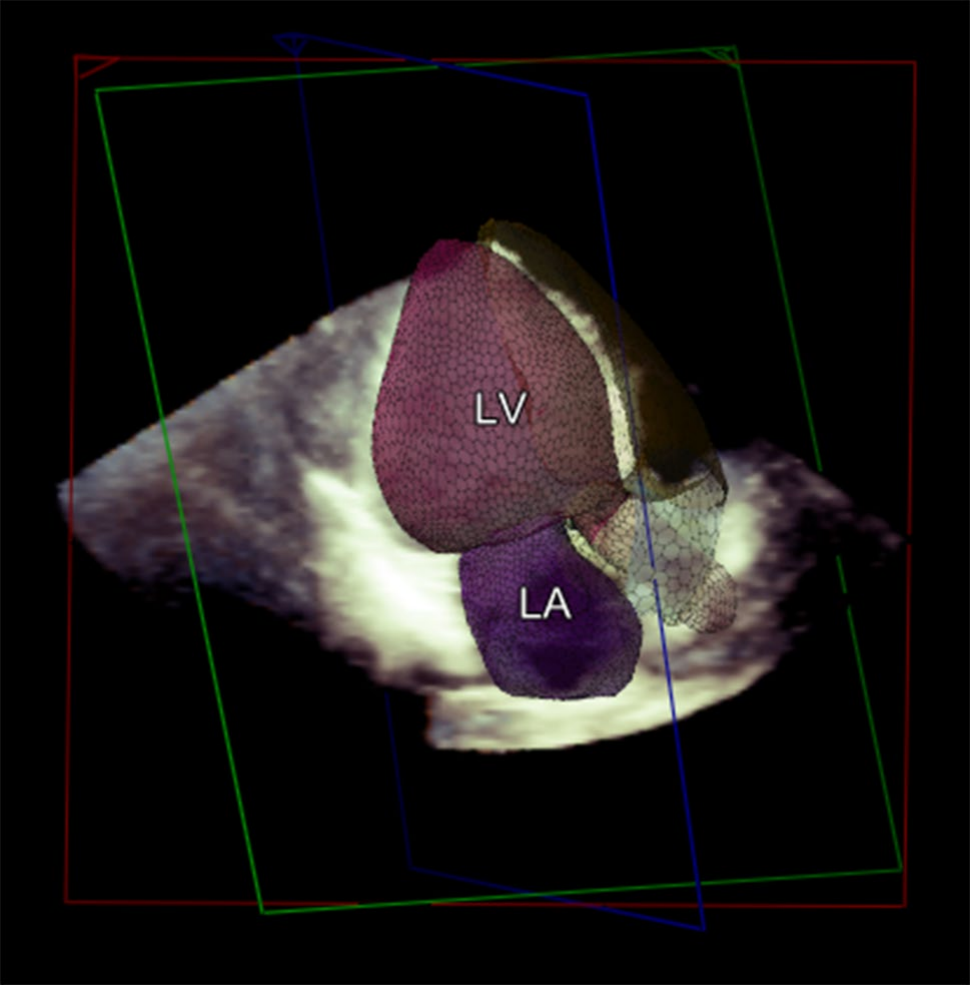
Example of a model created by the LHM. The model as generated by the LHM of left atrial (LA and left ventricular (LV) volumes

Automatic: Offline fully automatic analysis of the datasets was performed using the Q Lab advanced ‘Heart Model’ analysis software. This software has previously described in detail before by Tsang et al. [1, 2]. In brief, this software detects the endocardial surfaces by using an adaptive algorithm. This identifies a global end-diastolic shape which it uses in combination with motion detection to determine an end-systolic cavity [1, 2]. The program combines information from a database of 1000 3D TTE studies and its endocardial surface detection to model the LA and LV. Afterwards it matches features from the known datasets to the current patient for which it needs, much like manual measurements, a minimum of approximately 14 or 15 LV segments. The final model (Fig. 1) is displayed with the possibility to manually edit the contours if the user deems this necessary. To better estimate the added value over existing 3D techniques we chose not to manually edit the contours.

### Cardiac computed tomography

#### Data acquisition

Acquisition was performed on a 3rd generation dual-source CT (Somatom Force, Siemens Healthcare, Forchheim, Germany). Retrospective ECG gated spiral acquisition was used, with a mean dose length product (DLP) of (estimated effective dose 5 mSv, using k = 0.017) 362 mGy-cm kVp was modulated to patient size. Besides patient size and the selected kV the mA was modulated to the heartrate to provide a high mA pulse during 1–40% of the RR interval. The pitch was adapted to increase proportionally with higher heartrates. Reconstructions were made with a medium smooth kernel, with a slice thickness of 1.5 mm at an increment of 0.4 mm at every 5% of the RR interval.

#### Image processing

Images were analyzed semi-automatically using Syngo Via software (vb 10., Siemens, Forcheim Germany). Image analysis was performed by AC, with 1 year of cardiac CT experience. All cardiac phases were analyzed the software automatically detects the ED and ES phase, this was manually changed if needed. The endo and epicardial contours are automatically placed by the software and manually adjusted were need. The papillary muscles and if present trabeculations were included into the LV lumen. The basal plane was selected perpendicular to the short axis at the level of the mitral valve. Care was used to make sure the basal plane was on the same level in the ED and ES phase.

### Statistics

The IBM SPSS^®^ statistics 21.0 software was used to analyze the data. Continuous variables were presented as mean ± standard deviation (SD) or as median with an interquartile range. Categorical variables were presented as frequencies and percentages. We tested for normality by calculating Z-values of skewness and kurtosis, using the Shapiro–Wilk test and by visually assessing the data. For comparison of normally distributed continuous variables between two groups the student’s *t* test was used. To quantify correlations the Pearson or Spearman correlation test was applied. Intra-observer and inter-observer agreement between two investigators (A.T.H, J.S.M.) were assessed by repeated analysis of the same images in a third of the dataset a month after initial analysis at the same images and blinded to the initial results. The limits of agreement between two measurements were determined as the mean of the differences (bias) ± 1.96 SD and presented in a Bland–Altman plot [5]. Additionally, the coefficient of variation (COV) was provided to compare the dispersion of two variables. The COV was defined as either the SD of the differences of two measurements divided by the mean of their means. The statistical tests were two sided and a p-value below 0.05 was considered significant.

## Results

Fifty-three patients with a BAV were included; baseline characteristics are presented in Table 1. Eight patients also had Turner syndrome. Four patients previously underwent aortic coarctation resection and three patients underwent a balloon dilatation of their stenotic aortic valve. One patient underwent closure of a type II atrial septal defect.

**Table 1:** Baseline characteristics of the study population (n = 37)

Parameter	Median (IQR)
Baseline	
Men, n (%)	25 (68)
Age (years)	35.2 (23)
Height (cm)	178 (26)
Weight (kg)	72 (24)
BMI (kg/m^2^)	23.9 (3.0)
SBP (mmHg)	122 (20)
DPB (mmHg)	80 (19)
3DE LVEF < 50%, n (%)	16 (43)
Mitral valve	
E-wave (m/s)	0.70 (0.2)
A-wave (m/s)	0.50 (0.2)
E/A-ratio	1.2 (0.9)
DT (ms)	209 (67)
E’ septal, cm/s	7.8 (3.1)
Ee’-ratio	9.0 (3.6)
Aortic valve	
BAV (n = 32)	
No AoI, n= (%)	5 (16)
Mild AoI, n= (%)	21 (66)
Moderate AoI, n= (%)	5 (16)
Severe AoI, n= (%)	1 (3)
Peak velocity (m/s)	2.65 (1.6)
VT (cm)	52.8 (45)
Gradient (m/s)	28 (33)
TS* (n = 5)	
No AoI, n= (%)	4 (80)
Mild AoI, n= (%)	1 (20)
Moderate, AoI n= (%)	0 (0)
Severe AoI, n= (%)	0 (0)
Peak velocity (m/s)	1.4 (0.6)
VTI (cm)	28.4 (12)
Gradient (m/s)	8 (6.5)

Data are expressed as median and inter quartile range (IQR) or as ‘n=, (%)’ for the variables ‘gender’, 3DE LVEF<50% and ‘aortic insufficiency’

*AoI* aortic insufficiency, *BMI* body mass index, *SBP* systolic blood pressure, *DBP* diastolic blood pressure, *DT* deceleration time

*Subgroup of patients with Turner syndrome (TS) and a bicuspid aortic valve (BAV)

In all 53 patients, 2DE measurements could be performed and functional echo parameters were measured, as presented in Table 1. In four patients, no 3D dataset could be acquired. Additionally, 12 patients were excluded based on poor imaging quality.

### Left ventricle

#### Protocol 1: LHM versus conventional echocardiography

**Fig. 2:**
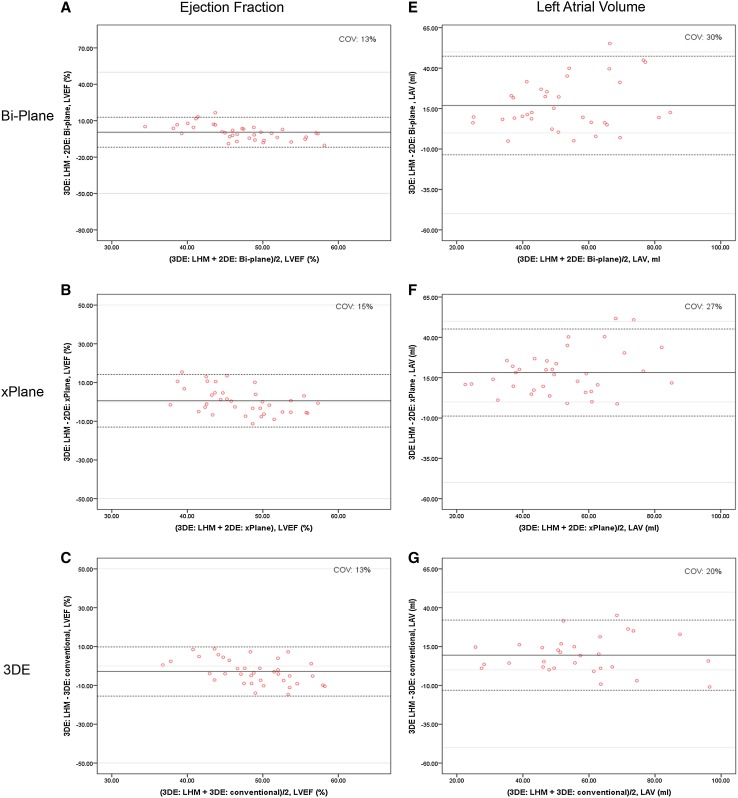
Bland–Altman plots demonstrating inter-modality agreement of EF and LAV in 2DE biplane (*panel* **a** and **e**), 2DE xPlane (*panel* **b** and **f**), 3DE (*panel* **c** and **g**) compared to the LHM. Ejection fraction measured by bi-plane 2DE (**a**), by xPlane 2DE (**b**) and by 3DE (**c**). Left atrial volume measured by bi-plane 2DE (**e**), by xPlane 2DE (**f**) and by 3DE (**g**). The* solid lines* depict the mean difference of the two measurements; the* dashed lines* depict the limits of agreement.* COV* coefficient of variation

There was a good correlation between the LHM and the manual bi-and xPlane measurements for LVEDV, LVESV, and LVEF. In Table 2 the measurements of LV volumes and function and LA maximal volume are presented for all different methods. Figure 2a, b show the Bland–Altman plots for EF by 2DE bi-plane and xPlane compared to the LHM. The results of the agreement analysis between the LHM and the measurements based on the 2DE are shown in Table 2. For clarity purposes, we did not include mutual correlations between the different echo modalities; however, Bi-plane and xPlane had a high correlation for LVEDV (r = 0.977), LVESV (r = 0.978) and LVEF (r = 0.702). Additionally, both methods correlated strongly with conventional 3D as expected.

**Table 2:** Correlations between LHM and four different methods of volumetric chamber quantification

Method	Phase	Mean, SD	Pearson’s r ^†^	Bias	Lower LOA	Upper LOA
LHM (n = 37)						
	EDV (ml)	146 ± 48				
	ESV (ml)	77 ± 29				
	EF (%)	47 ± 5				
	LA (ml)	61 ± 19				
LHM 2nd dataset (n = 12)						
	EDV (ml)	143 ± 61	0.99**	−0.8	−9	7
	ESV (ml)	78 ± 34	0.99**	1	−7	9
	EF (%)	45 ± 4	0.58*	−1.5	−7	4
	LA (ml)	57 ± 19	0.98**	−1	−9	7
2DE Bi-plane (n = 37)						
	EDV (ml)	145 ± 54	0.93**	0.06	−41	41
	ESV (ml)	77 ± 31	0.88**	0.2	−29.1	29.4
	EF (%)	47 ± 8	0.63**	0.5	−11.8	12.8
	LA (ml)	43 ± 16	0.61**	17	−13.6	47.4
2DE xPlane (n = 37)						
	EDV (ml)	143 ± 52	0.94**	2	−32	36.4
	ESV (ml)	77 ± 30	0.88**	0.8	−27.1	28.8
	EF (%)	47 ± 8	0.43**	0.6	−13	14.2
	LA (ml)	42 ± 16	0.69**	18	−8.8	45.2
3D (n = 37)						
	EDV (ml)	143 ± 50	0.97**	2	−23	27.6
	ESV (ml)	71 ± 26	0.91**	6	−16.9	29.8
	EF (%)	50 ± 7	0.51**	−3	−15.5	9.8
	LA (ml)	53 ± 19	0.81**	9	−13.1	32.1
CT (n = 37)						
	EDV (ml)	185 ± 63	0.88**	−42	−102.3	18.9
	ESV (ml)	67 ± 24	0.81**	10	−24.1	43.6
	EF (%)	64 ± 5	0.24	16	−28.3	−4.7

Data are presented as mean and SD

*EDV* end-diastolic volume,* ESV* end-systolic volume,* EF* ejection fraction,* LA *left atrium,* LOA* limit of agreement,* COV *coefficient of variation

^†^Compared with the LHM, **p<0.01, *p<0.05. A negative mean implies a smaller value was given by the LHM

The LHM correlated strongly with the 3D LV measurements as shown in Table 2. The LHM seems to estimate slightly larger LVEDV and LVESV and a smaller LVEF compared to manual 3D (Table 2). Figure 2c shows the Bland–Altman plot for EF measured by 3DE. 16 patients (30%) had to be excluded due to poor imaging quality, which is considered acceptable in a routine clinical setting for 3DE.

#### Protocol 2: LHM versus CT

**Fig. 3:**
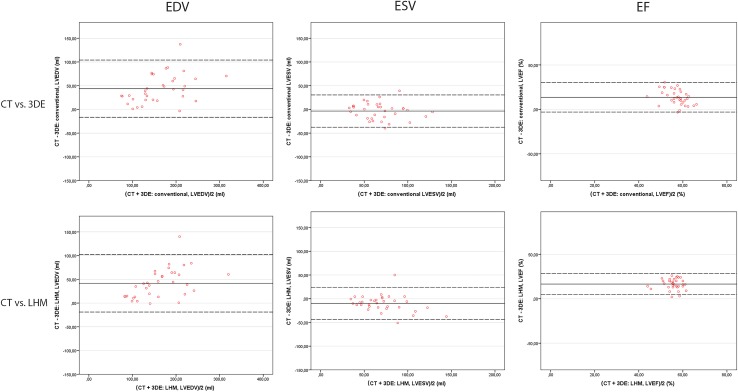
Bland–Altman plots demonstrating inter-modality agreement between CT and 3DE and LHM. Agreement in end-diastolic (EDV) and end-systolic volume (ESV) and ejection fraction (EF) comparing CT versus conventional 3D echocardiography (*top row*) and CT versus the LHM (*bottom row*)

**Fig. 4:**
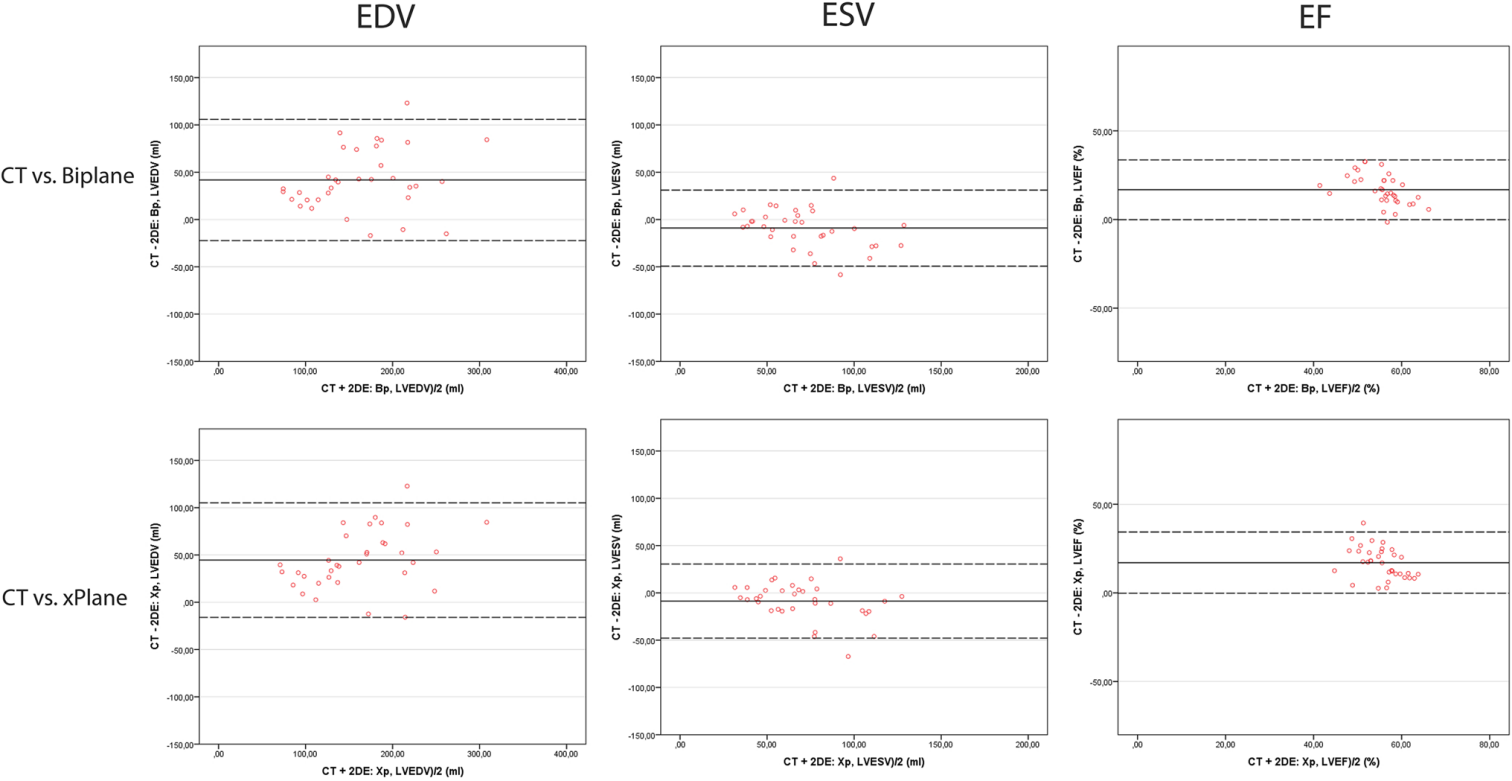
Bland–Altman plots demonstrating inter-modality agreement between CT and 3DE and LHM. Agreement in end-diastolic (EDV) and end-systolic volume (ESV) and ejection fraction (EF) comparing CT versus 2D bi-plane echocardiography (*top row*) and CT versus xPlane echocardiography (*bottom row*)

In the second protocol, where CT was used as a golden standard method, agreement was found for EDV and ESV (r = 0.88 and r = 0.81); mean differences were 42 ml (23%) and 10 ml (15%) respectively (p < 0.001 and p = 0.002). However, compared to CT the LHM seems to estimate smaller LVEDV (Table 2) and consequently also produces a smaller LVEF (mean difference 16%, p < 0.001) as shown by the positive difference in EF between CT and the LHM in Fig. 3. The correlations for Bi-plane, xPlane and conventional 3D with CT for LVEDV, LVESV and LVEF (Fig. 4) were comparable; a relatively small LVEDV and therefore a lower LVEF compared to CT. Three patients did not undergo a CT scan for personal reasons, those three patients are excluded from analysis.

#### Inter- and intra-observer variability

The LHM has no intra-, or inter-observer variation as the model produced the exact same measurements when the same dataset is used (mean difference: 0 ± 0, p < 0.001). In addition, when the measurements were repeated on a second acquisition of the same patients (n = 12) there was some variation but generally there was good agreement reflected by a small bias and narrow limits of agreement (Table 2). The inter-observer variability for the LVEDV and LVESV on conventional 3DE was calculated (r = 0.909, p < 0.001 and r = 0.862, p < 0.001) in 14 (38%) patients.

### Left atrial volume

The LAV was estimated consistently significantly larger by the LHM (Table 2) compared with on both 2DE and 3DE as is shown in Fig. 2e–g. This ‘overestimation’ was the most evident comparing to the 2D methods and less pronounced compared with manual 3D measurements. The correlation between 2D Bi-plane and 2D xPlane measurements of the LA volume was very high (r = 0.946, p < 0.001). When comparing the LAV measured on 2D with the LA volume measured on 3D, xPlane outperforms Bi-plane (r = 0.632, p < 0.001 and r = 0.551, p = 0.002 respectively).

## Discussion

The main findings of this study can be summarized as follows:

- Automated chamber quantification is feasible in patients with bicuspid aortic valve disease in a routine clinical setting.
- The ‘Heart Model’ provides accurate automatic measurements of LVEDV, LVESV and LVEF and LAV compared to 2D and 3D echocardiography.
- There is no inter or intra-observer variability and very little ‘inter-dataset’ variability.

### Left ventricular assessment

In daily clinical practice LV function is routinely estimated by bi-plane Simpson method of disk-summation; however, this requires sufficient experience and has limitations in accuracy and reproducibility, especially due to the geometric assumptions of the shape of the ventricular or atrial cavity inherent to 2DE [4]. Moreover, the lack of a third dimension is generally considered to result in high inter-measurement variability and limits endocardial visualization, predominantly of apical lateral segments. Foreshortening of the LV often performed in an attempt to alleviate this shortcoming causes reduced accuracy and reproducibility [6]. Volumetric quantification from 3D data sets allows frame-by-frame detection of endocardial surface and does not require manual image plane positioning or geometric assumption [7] and has furthermore been shown to have a higher reproducibility than 2DE [8–10].

The bias of CT and 3D echocardiography is approximately as low as the bias of MRI when estimating LVEF [11]. Additionally, previous studies showed that for LVEF CT has the best correlation with MRI. In this light it is remarkable that in our study the correlation with CT is weak, as the EDV seems to be systematically estimated to be larger by CT than by the LHM or other echo modalities [11, 12]. This systematic underestimation may in part reflect an inter observer variability, as more of the trabeculations were included in the LV cavity for CT, entailing a larger EDV. Also, the trabeculations in this population may be more pronounced than in healthy subjects, adding to the observed difference.

Left heart chamber quantification by 3DE is hampered by several factors, mainly the ease with which these techniques can be used in daily clinical practice as a degree of experience is required. However, especially in the growing and aging population of patients with valvular and congenital heart disease, where patients regularly undergo extensive echocardiographic evaluation, user-independent and non-invasive follow-up imaging is much needed.

### Added value of LHM

Accurate and reproducible measurements of left chamber volumes are very important in clinical practice, as they correlate with prognosis and determine treatment strategies. Moreover, in order to test new therapies, changes in these parameters must be accurate to demonstrate the efficacy of medical therapy or intervention. Our results indicate that intra-observer, inter-observer, and test–retest reproducibility of this method are very high and even exceptionally for intra-observer variability (0%) since this algorithm has no human interaction (i.e., phase selection and contouring are automatic).

We had to exclude 16 patients (30%) in total due to imaging quality limitations which is comparable to conventional 3DE in a routine clinical setting.

The main concern with 3D echocardiography is the accessibility in terms of time and skill required to produce accurate and reproducible results. The algorithm described in this study promises to improve on these points. This study demonstrates a high feasibility and accuracy in a population of patients with BAV disease, a population where fast, reliable, user-independent and non-invasive follow-up imaging by echocardiography is imperative.

### Left atrial volume

Another remarkable finding is the relatively large LA volume estimated by the LHM compared to 2DE measurements. First, we suggested that this could be explained by the inclusion of the pulmonary vein orifice into the left atrial cavity by the algorithm. However, when carefully re-evaluating the LA contours, most seemed to adequately follow the left atrial walls. Another explanation could be that it is actually an underestimation of the 2DE measurements. This can partly be explained by the use of the length area formula which assumes the LA to be ellipsoid, which is evidently not always the case [4]. The correlation of LA volume measurements with 3D TTE was better. Still, inherent flaws of 2D imaging in combination with LA anatomy could contribute to this discrepancy. In the standard 2DE the LA measurements are performed on the A4C and A2C views which are focused on the true long axis of LV. The true long axis LA may not be in the same plane as the LV and therefore may appear foreshortened on the apical four chamber view. In xPlane a special focus view on the true LA long axis was acquired which better correlated with the LHM.

### Limitations

The golden standard for quantitative volumetric heart chamber assessment is cardiac magnetic resonance imaging (MRI). Unfortunately, no cine cardiac images were available in this study. Therefore, no comparison with MRI could be made; consequently, we used volumetric data from CT as an additional source of validation. Additionally, we feel that EDV by 2DE has been slightly underestimated and therefore 3DE is best used as a reference modality. We were strict when it came to image quality therefore we had to exclude 12 patients from analysis. However, a sufficiently large cohort could be analyzed.

## Conclusion

Automated chamber quantification is feasible and accurate in patients with bicuspid aortic valve disease in a routine clinical setting. The ‘Heart Model’ provides accurate automatic measurements of LVEDV, LVESV and LVEF and LAV and has a high reproducibility between dataset and no inter or intra-observer variability. It does however seem to underestimate end-diastolic volume compared to CT.
